# Design and characterization of calprotectin tetramerization variants for probing the role of oligomerization in receptor activation

**DOI:** 10.1002/pro.70399

**Published:** 2025-12-22

**Authors:** Velia Garcia, Areetha D'Souza, Natalia Kozlyuk, Yasiru R. Perera, Steven M. Damo, Walter J. Chazin

**Affiliations:** ^1^ Department of Chemistry Vanderbilt University Nashville Tennessee USA; ^2^ Center for Structural Biology Vanderbilt University Nashville Tennessee USA; ^3^ Department of Biochemistry Vanderbilt University Nashville Tennessee USA; ^4^ Department of Life and Physical Sciences Fisk University Nashville Tennessee USA

**Keywords:** calcium‐binding, calprotectin, NMR, oligomerization, S100 proteins, small‐angle x‐ray scattering (SAXS), x‐ray crystallography

## Abstract

Calprotectin is a heterodimer of the S100A8 and S100A9 EF‐hand calcium binding proteins, which activates cell surface receptors that signal through the NF‐κB inflammatory signaling pathway. Like all S100 proteins, calcium‐induced conformational changes in calprotectin are required for binding to partner proteins. In the case of calprotectin, the addition of calcium correlates with the formation of a dimer of heterodimers (heterotetramer). Ligand‐induced receptor oligomerization has been proposed as a mechanism of receptor activation. Conversely, it has also been suggested that calprotectin tetramerization can inhibit binding to receptors and serve as an autoinhibitory mechanism. In order to investigate the biological relevance of calprotectin tetramerization and facilitate in‐depth biophysical and structural analysis, we have prepared three tetramerization‐deficient variants: two single‐site S100A8 mutations of hydrophobic isoleucine residues mediating the tetramer interface to lysine (I60K, I73K) and the corresponding double‐site mutant (I60K/I70K). Dynamic light scattering, small‐angle x‐ray scattering, and nuclear magnetic resonance spectroscopy showed that all three tetramer‐deficient variants remain as dimers in solution even in the presence of 40‐fold excess calcium and undergo calcium‐induced conformational changes. The crystal structure of I73K was determined to atomic‐level resolution and confirms that the mutations cause only subtle, localized effects on the structure. Together, the results indicate that these tetramerization‐deficient mutants will be useful reagents for discerning the functional role of calprotectin oligomerization in the activation of inflammatory receptors.

## 1 INTRODUCTION

Inflammation is an essential biological process that serves as an important reaction to cell stress, injury, or infection (Medzhitov, 2021; Soliman & Barreda, 2023). However, chronic or misregulated inflammation can lead to tissue damage and is associated with a number of diseases. Inflammation is promoted externally by pathogen‐associated molecular pattern molecules and internally by damage‐associated molecular pattern molecules (DAMPs) (Newton & Dixit, 2012; Roh & Sohn, 2018). The S100 protein calprotectin is a highly abundant DAMP in innate immune cells that promotes inflammation through direct interaction with cell‐surface receptors, including the receptor for advanced glycation end‐products (RAGEs), toll‐like receptor 4 (TLR‐4), and cluster of differentiation 33 (CD‐33). However, biophysical and structural analyses of the physical interactions of these receptors with calprotectin are limited.

S100 proteins constitute a class of EF‐hand calcium‐binding proteins with over 20 members (Heizmann et al., 2002). All S100 proteins are comprised of paired EF‐hand motifs and with one exception (S100G) form obligate dimers mediated by hydrophobic residues that form an integrated hydrophobic core (Potts et al., 1995). Structures of a number of S100 proteins have been determined in the absence and presence of calcium (Dempsey et al., 2012; Mäler et al., 2002; Moroz et al., 2002). These revealed that unlike prototypical EF‐hand calcium‐binding proteins such as calmodulin, S100 proteins undergo a modest conformational change upon calcium binding: a shift in the C‐terminal EF‐hand that changes the orientation of Helix 3. This change in conformation however is critical to increased affinity for targets (Bhattacharya et al., 2004). S100 proteins are also distinguished by two transition metal binding sites that are formed at symmetrically disposed sites in the dimer interface (Gilston et al., 2016).

Calprotectin is composed of two S100 proteins, S100A8 and S100A9, which are unique because they preferentially form heterodimers (Hunter & Chazin, 1998). In addition to binding calcium ions, calprotectin binds zinc, copper, manganese, nickel, and iron (II) ions with high affinity at its two distinct transition metal binding sites (Damo et al., 2013; Nakashige et al., 2017). A unique characteristic of calprotectin is that the calcium‐induced conformational change correlates with the self‐association of the protein to a specific tetrameric state, that is, a dimer of the heterodimer (Korndörfer et al., 2007). Binding of transition metals is also associated with oligomerization but this is less well studied. In the case of zinc, the effect appears to be less specific as it leads to the aggregation of calprotectin in vitro (Perera et al., 2025). It is unclear whether oligomerization plays a role in receptor activation or other biological functions.

Tetramerization/oligomerization of calprotectin has been proposed to play an autoinhibitory role in receptor interaction (Russo et al., 2022; Vogl et al., 2018). However, these studies were based on mutations that suppressed calcium binding and comparisons to the S100A8 and S100A9 homodimers, approaches that do not enable direct separation of function analysis of the role of tetramerization in calprotectin function. An alternative, direct strategy involves disrupting tetramerization by mutating residues that play key roles at the interface between the two heterodimers in the tetramer. This was first demonstrated for a variant with Ile60 in S100A8 mutated to Lys (I60K), which was shown to disrupt the formation of the tetramer (Silvers et al., 2021; Stephan & Nolan, 2016).

To extend the analysis of calprotectin tetramerization, we first performed an in‐depth analysis of the x‐ray crystal structure of the calcium‐loaded tetramer (Krissinel, 2010). This revealed that the interface between the two heterodimers in the tetramer is stabilized by multiple hydrophobic interactions with a particularly large interaction surface between the two S100A8 subunits. Based on these insights, we designed two new tetramer‐deficient variants with S100A8 Ile to Lys mutations (I73K and I60K/I73K). Dynamic light scattering (DLS), small‐angle x‐ray scattering (SAXS), x‐ray crystallography, and nuclear magnetic resonance (NMR) spectroscopy were used to characterize the structures and oligomeric states of these variants along with I60K in the presence and absence of calcium. All variants bound calcium and retained the structure of calprotectin but did not form tetramers, indicating they can serve as useful reagents for discerning the functional role of oligomerization in the activation of cell‐surface receptors.

## 2 RESULTS

It is well established that at concentrations in the nM range or higher, the cysteine residues in S100A8 (Cys42) and S100A9 (Cys3) will form cross‐linked disulfides (Hunter & Chazin, 1998). Calprotectin incorporating the corresponding cysteine to serine mutations has been extensively analyzed by biophysical and structural techniques; these studies showed that the structure is essentially identical to wild‐type (WT) calprotectin and that there are no significant effects on its antimicrobial activity (Damo et al., 2013; Hunter & Chazin, 1998; Perera et al., 2025). To confirm that CP* interacts with its receptors, we used isothermal titration calorimetry to measure its affinity for the VC1 tandem domains of RAGE (Figure S1, Supporting Information), which returned a K_d_ value of 3.8 ± 4.4 × 10^−7^ M. This variant has been used in all studies reported herein and is referred to as CP*.

### 2.1 Calcium induces changes in calprotectin oligomerization state

As noted above, binding of calcium by calprotectin is associated with the formation of a tetrameric state. In the effort to develop reagents to investigate the functional importance of tetramerization, DLS and NMR spectroscopy were used to comprehensively characterize the oligomerization state of CP* and monitor calcium‐induced changes in the structure. DLS measures the diffusion behavior of particles in solution and is a versatile method to obtain information on particle size, shape, and aggregation states in the concentration range from ~100 μM to below 1 nM. Although DLS cannot resolve a rapidly exchanging mixture of dimer and tetramer as two distinct peaks, comparison of the extent of polydispersity provides an effective means to distinguish if a solution contains a single or multiple species.

**FIGURE 1:**
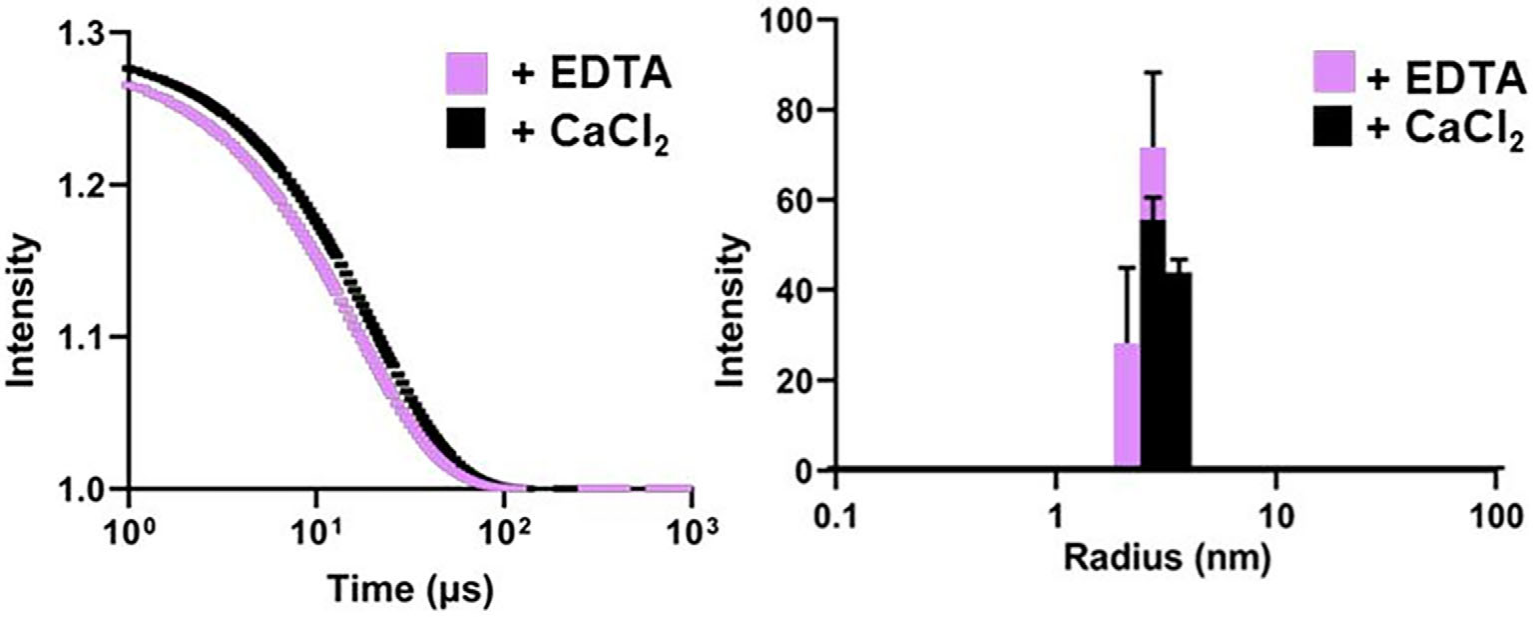
Calcium‐induced tetramerization of CP* monitored by DLS. (Left panel) Autocorrelation function of 50 μM CP* in the absence of calcium (purple) and in the presence of excess (2 mM) calcium (black). (Right panel) Histogram representation of the data. The derived radius increases from ~2.8 to ~3.2 nm; the polydispersity index of 12.2% and 13.5% for both samples respectively indicates the solutions are monodisperse.

DLS was analyzed for samples of CP* in the absence and presence of calcium. CP* in the absence of metals yielded a hydrodynamic radius of ~2.8 nm and a polydispersity percentage of less than 15, consistent with a homogeneous dimer (Figure 1). Upon the addition of calcium, a modest increase in hydrodynamic radius to ~3.3 nm is detectable in both the intensity autocorrelation function and calculated histogram. Over the course of a calcium titration, a mixture of CP* dimer and tetramer is observed, which is reflected in a polydispersity >15%. However, in the presence of a large excess of calcium (2 mM) the data are consistent with a homogeneous tetrameric state (Figure 1). Transition into higher‐order oligomeric states has been reported for S100 proteins under conditions of excess calcium, but this is not observed for CP*. The requirement of high levels of calcium to drive the protein fully into the tetrameric state is presumably a byproduct of weak affinity for calcium in the N‐terminal S100‐specific calcium binding site of S100A8, which has an Asp residue in place of the critical, highly conserved Glu at the C‐terminal end of the calcium binding loop.

**FIGURE 2:**
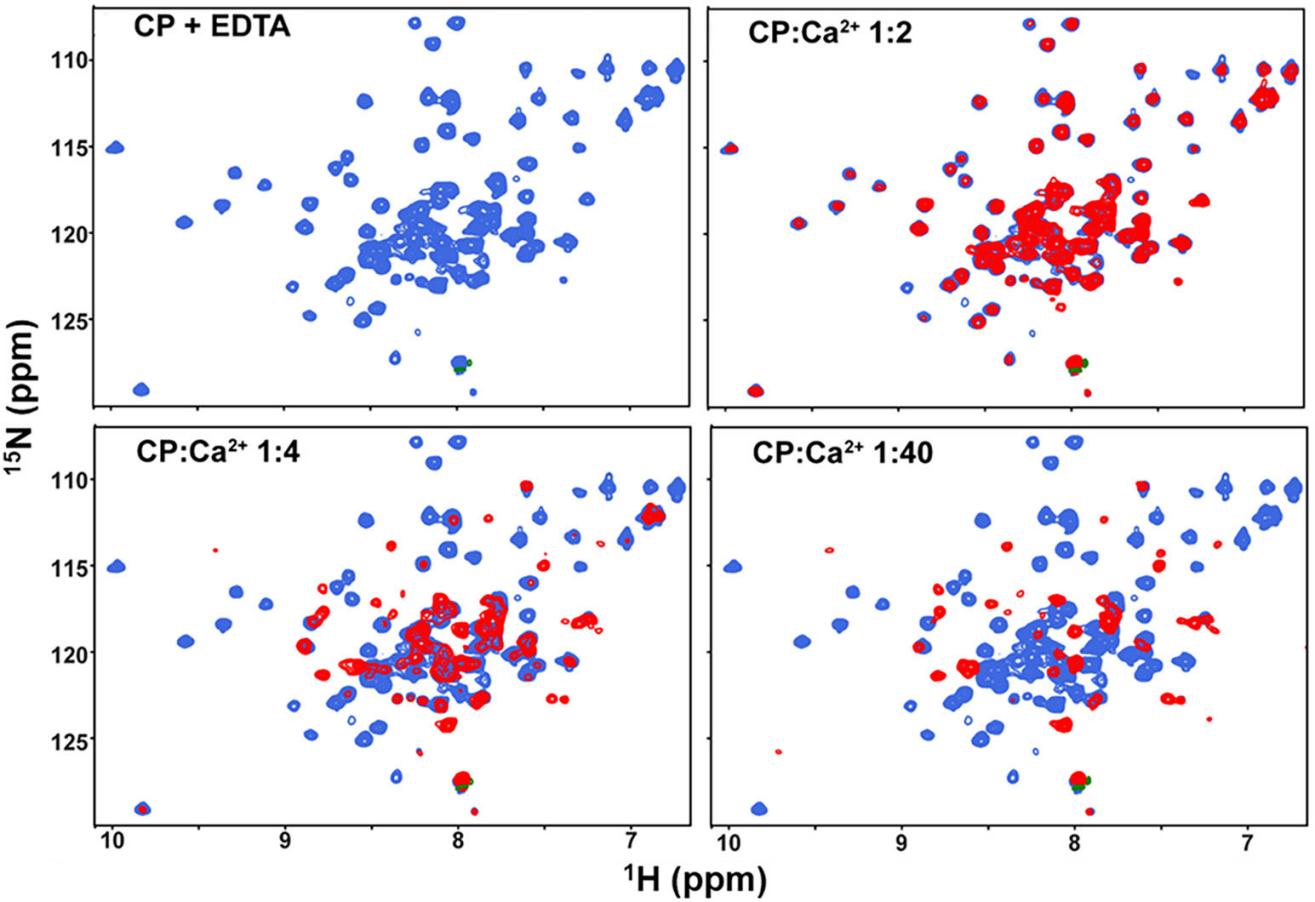
Conversion of the 24 kDa CP* dimer to the 48 kDa tetramer. Two dimensional ^15^N‐^1^H HSQC NMR spectra at 25°C of the selectively ^15^N‐enriched S100A9 subunit of CP* obtained at 600 MHz in the absence (blue) and presence (red) of increasing concentrations of calcium.

NMR spectroscopy was used to further characterize the oligomerization state as well as the conformational changes induced by the binding of calcium. Since calprotectin is a heterodimer and the S100A8 and S100A9 subunits are expressed separately, it is possible to isotopically enrich just one subunit to simplify the NMR spectrum. Two‐dimensional ^15^N‐^1^H HSQC NMR spectra were acquired in the absence and presence of calcium for samples generated from ^15^N‐S100A9 and unlabeled S100A8. All samples were treated with EDTA to ensure the removal of calcium and transition metals at the starting point. The NMR spectrum obtained in the absence of metals showed the expected relatively narrow linewidths and excellent dispersion of signals consistent with a well‐folded 24 kDa S100 protein dimer (Figure 2). As anticipated based on the DLS results, progressive line broadening is observed as calcium is added to the solution, which we attribute to exchange between the 24 kDa dimer and 48 kDa tetramer. The increase in linewidth is accompanied by a significant reduction in S/N as the titration proceeds, a reflection of the slower tumbling in solution and more rapid relaxation of the tetramer. At the 1:40 ratio of calcium ions to CP*, the bulk of the signals are broadened beyond detection and only signals from disordered regions and flexible side chain amides are observed (Figure 2), consistent with the slow tumbling of a 48 kDa particle.

### 2.2 CP* tetramer is stabilized by interactions between all four subunits

**FIGURE 3:**
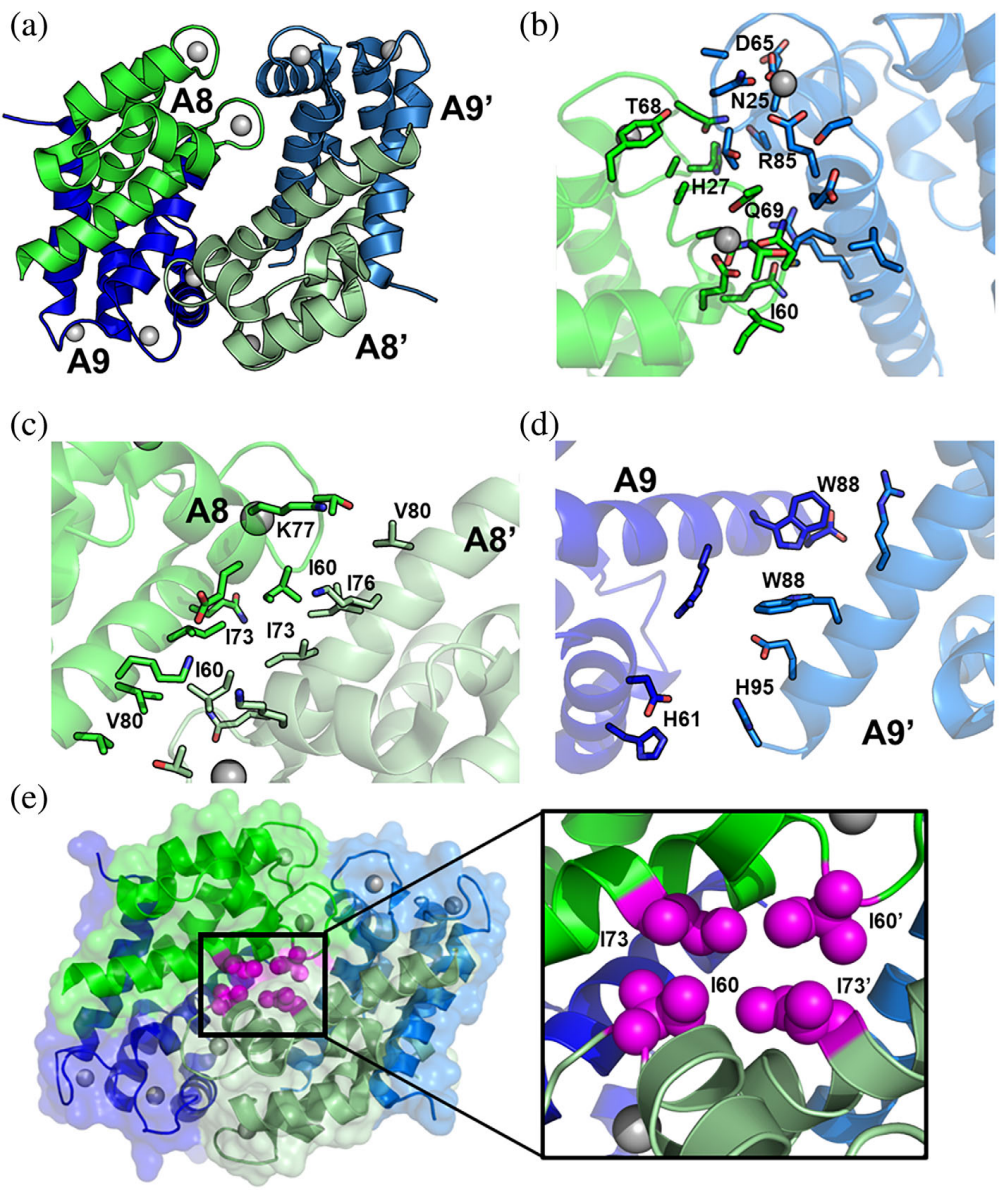
Analysis of the CP* tetramer interfaces. (a) Ribbon diagram of calcium‐loaded CP* tetramer (PDB ID: 1XK4). The S100A8 subunits are colored green and muted green and the S100A9 subunits blue and muted blue. Calcium ions are depicted as gray spheres. (b) Close up view of the A8/A9′ interface. Highlighted residues were identified by PDBePisa as stabilizing this interface. (c, d) Close up view of the A8/A8′ interface (c) and A9/A9′ interface (d) with residues identified by PDBePisa as stabilizing the corresponding interfaces. (e) CP* tetramer ribbon diagram and surface representation with residues I60 and I73 on A8 depicted as spheres. The inset zooms in on the inter subunit interface between I60 and I73.

A primary goal of this work was to design variants of CP* that would bind calcium and undergo the calcium‐induced conformational change but not form tetramers. We began by using the PDBePisa server to analyze the interactions that stabilize the tetramer in the x‐ray crystal structure of calcium‐loaded CP* (PDB 1XK4) (Krissinel, 2010). All four subunits are involved (Figure 3a). To distinguish the four subunits of the tetramer one CP* heterodimer is labeled A8/A9 and the other A8'/A9'.

The largest contributions to the tetramer interface are from contacts between A8/A9' and A8'/A9. The solvent accessible surface area (SASA) buried for each of these two interfaces is ~400 Å^2^ with the bulk of the interactions involving the calcium binding loops (Figure 3b). These two interfaces are stabilized by eight hydrogen bonds and two salt bridges (Table S1). Interestingly, key residues that stabilize this tetramer interface also directly chelate a calcium ion, including S100A8 residues N61 and E70 in the canonical EF‐hand binding site. The involvement of these residues is intriguing because they contribute to the beta sheet‐type interactions and hydrogen bonds between the two calcium binding loops that are integral to the well characterized allostery between paired calcium binding sites in EF‐hand proteins (Mäler et al., 2002).

Contacts between Helices 3 and 4 of S100A8 and S100A8' also contribute significantly to the stabilization of the tetramer. Although the buried SASA of this interface is somewhat smaller at 325 Å^2^, it is remarkable because it contains no hydrogen bonds or salt bridges and involves only hydrophobic interactions. The key contacts involve residues I60, I73, I76, K77, and V80 (Figure 3c). Contacts between S100A9 and S100A9' are the fewest in number and bury a SASA of only 84 Å^2^. The key residues involved include H61, W88, and H95, and again, no hydrogen bonds or salt bridges are detected (Figure 3d). Intriguingly, H95 is part of the His6 transition metal binding site.

As noted above, our objective was to generate one or more separation of function variants that only affect tetramerization, while retaining all other properties. Considering all of the information gleaned from the analysis of the tetramer interfaces, our variant design focused on the specific residues contributing most to the stabilization of the tetramer based on the analysis of buried solvent accessible surface. These are in the interface between S100A8 and S100A8′: I60, I73, and I76. In seeking to make the minimal number of mutations, we selected I60 and I73 because they are close to each other with nearest interatomic distances of 6.3 and 4.5 Å, respectively (Figure 3e). Previous studies of the CP* variant with I60 mutated to lysine (I60K) using analytical SEC chromatography and analytical ultracentrifugation revealed it suppressed calcium‐induced tetramerization (Silvers et al., 2021; Stephan & Nolan, 2016). Therefore, we decided to generate this variant as well as I73K and I60K/I73K.

### 2.3 CP* tetramerization‐suppressing variants remain dimeric in the presence of calcium

**FIGURE 4:**
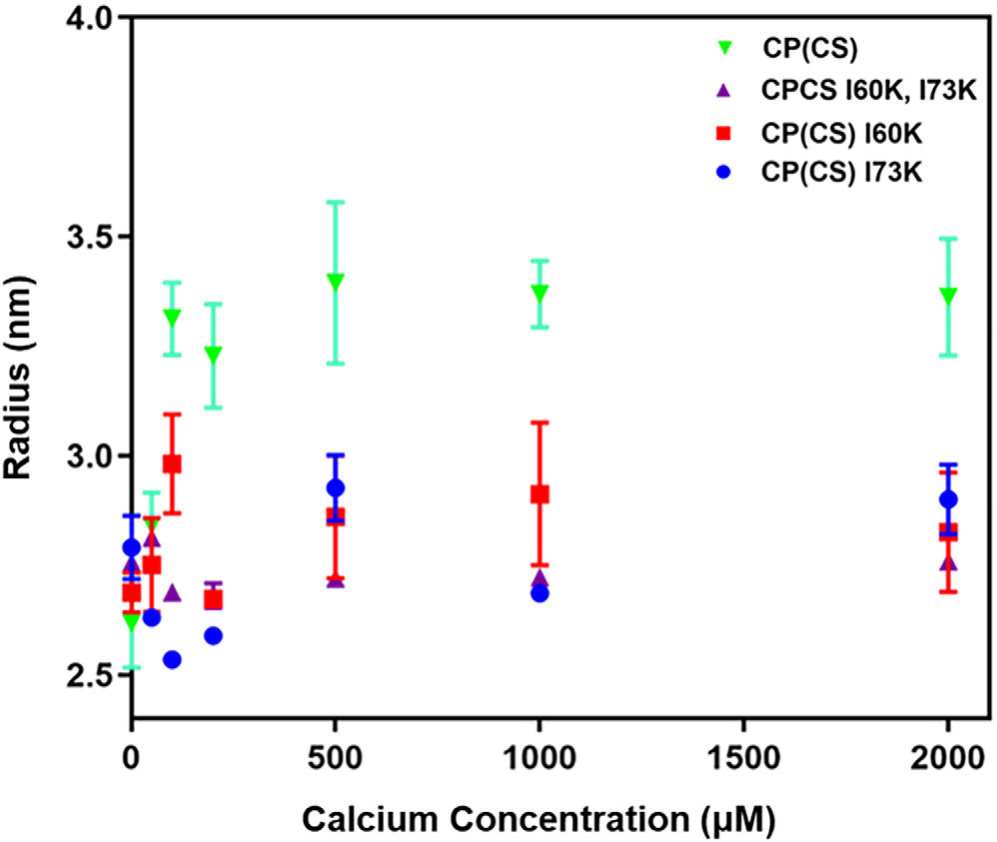
CP* tetramer‐deficient variants remain as dimers in solution. Calcium titration of CP* and CP* variants monitored by DLS. Experiments were performed at a protein concentration of 50 μM and titrated with CaCl_2_ up to 2 mM (40‐fold excess).

The three CP* variants containing S100A8 I60K, I73K and I60K, I73K mutations were expressed, purified, and their oligomerization states characterized by DLS. The hydrodynamic radius of each variant was measured over the course of a calcium titration and compared with the results obtained for the WT protein (Figure 4). Because calcium concentration in the extracellular milieu is on the order of ~1 mM, it is important that the tetramer‐deficient variants remain dimeric even at very high calcium concentrations. Therefore, the titration was extended well beyond the stoichiometric amount (100 μM calcium ions/CP* subunit) to a 40‐fold excess. In the absence of calcium, a hydrodynamic radius less than 3 nm was measured for all three variants, as was observed for CP*. The radius of all three tetramer‐deficient variants stayed below 3 nm even after the addition of excess calcium, indicating they remain in a dimeric state. This contrasts with CP*, for which an increase in radius to ~3.4 nm is already evident at one molar equivalent of calcium. Together, these results provide strong support for the hypothesis that the two designed calprotectin variants as well as the previously studied I60K are tetramerization‐deficient.

### 2.4 Tetramerization‐deficient variants do not perturb the native structure of CP* or the calcium‐induced conformational change

**FIGURE 5:**
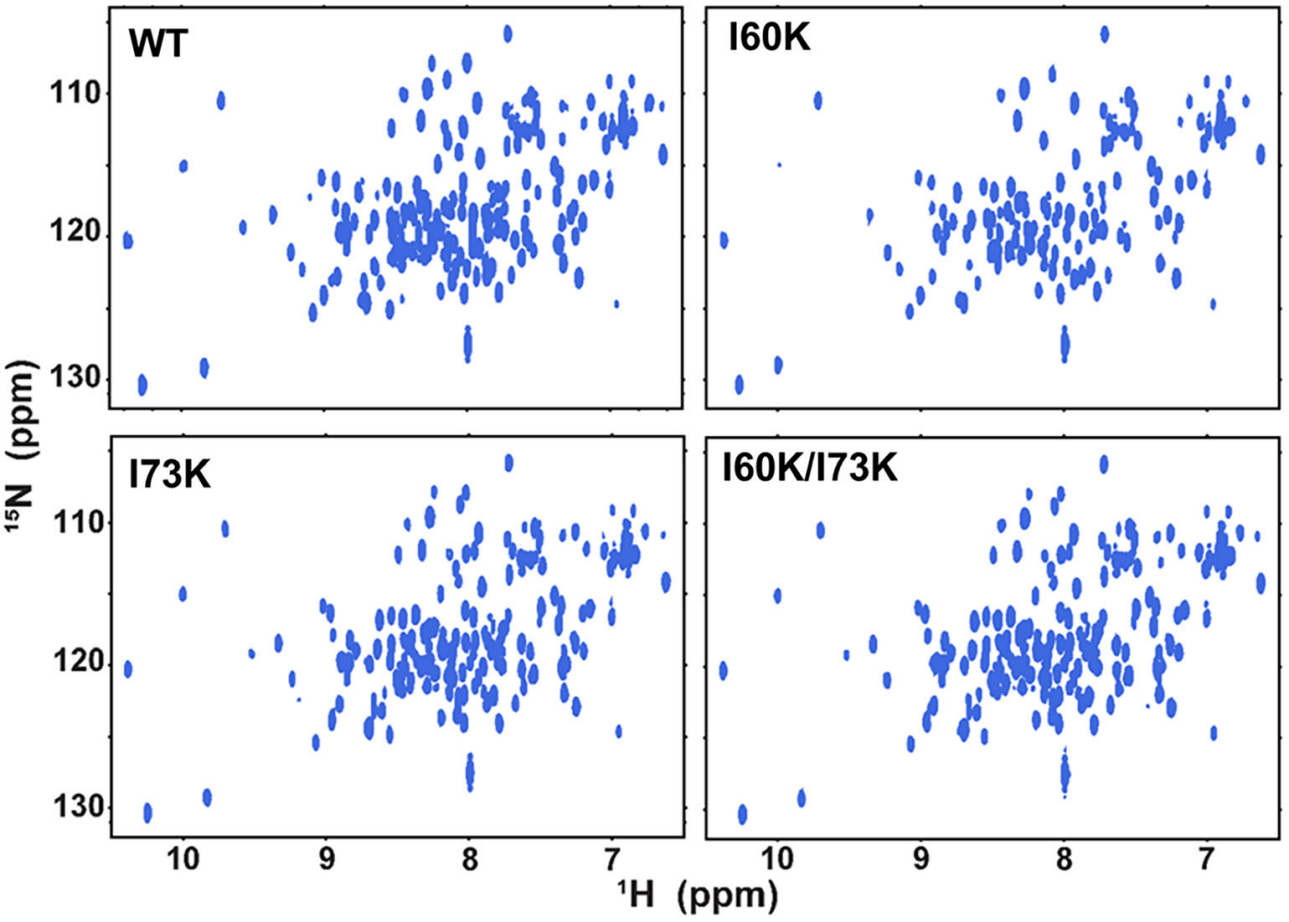
The Ile‐Lys mutations do not alter the structure of CP*. Two dimensional ^15^N‐^1^H NMR spectra of CP* and tetramerization‐deficient variants acquired at 900 MHz and 37°C at a protein concentration of 100 μM in the absence of calcium. All spectra show well‐dispersed peaks with homogeneity in peak shape and intensity, characteristic of well‐folded proteins.

NMR was used to test if the variants altered the structure of CP* or the conformational changes induced by the binding of calcium. To this end, samples of CP* and the three tetramer‐deficient mutants were produced with both subunits ^15^N‐enriched and 2D ^15^N‐^1^H HSQC NMR spectra acquired in the absence and presence of Ca^2+^ ions. Comparison of the spectra obtained in the absence of calcium confirmed that the overall structure is not affected by the Ile‐Lys mutations (Figure 5). The spectra of the three variants show good dispersion and a uniform distribution of symmetric peaks of the same shape and intensity, characteristic of a well‐folded protein, as is observed for CP*. In addition, the spectra of all three variants are nearly identical to that of CP* with only limited chemical shift perturbations, consistent with the limited number of mutations.

The NMR approach was also used to determine if the variants exhibit the characteristic conformational changes induced by the binding of calcium ion. S100 proteins undergo distinctive conformational changes upon binding of calcium that result in a change in the packing of Helix 3. Because NMR is very sensitive to alterations in chemical environment, NMR has been used extensively to monitor and characterize conformational changes in S100 proteins induced by the binding of calcium (Drohat et al., 1998; Harrison et al., 2023; Skelton et al., 1992). A large number of resonances affected upon calcium binding arises because the reorganization of the packing of Helix 3 alters its contacts with Helices 2 and 4 and in the case of calprotectin, additional contacts are generated in forming the tetramer. In the absence of resonance assignments, it is not possible to assign specific changes in structure. However, the extensive body of data on the calcium‐induced changes in NMR spectra of S100 proteins can be used to qualitatively assess if the spectral changes induced by calcium binding for CP* reflect those observed for other S100 proteins.

**FIGURE 6:**
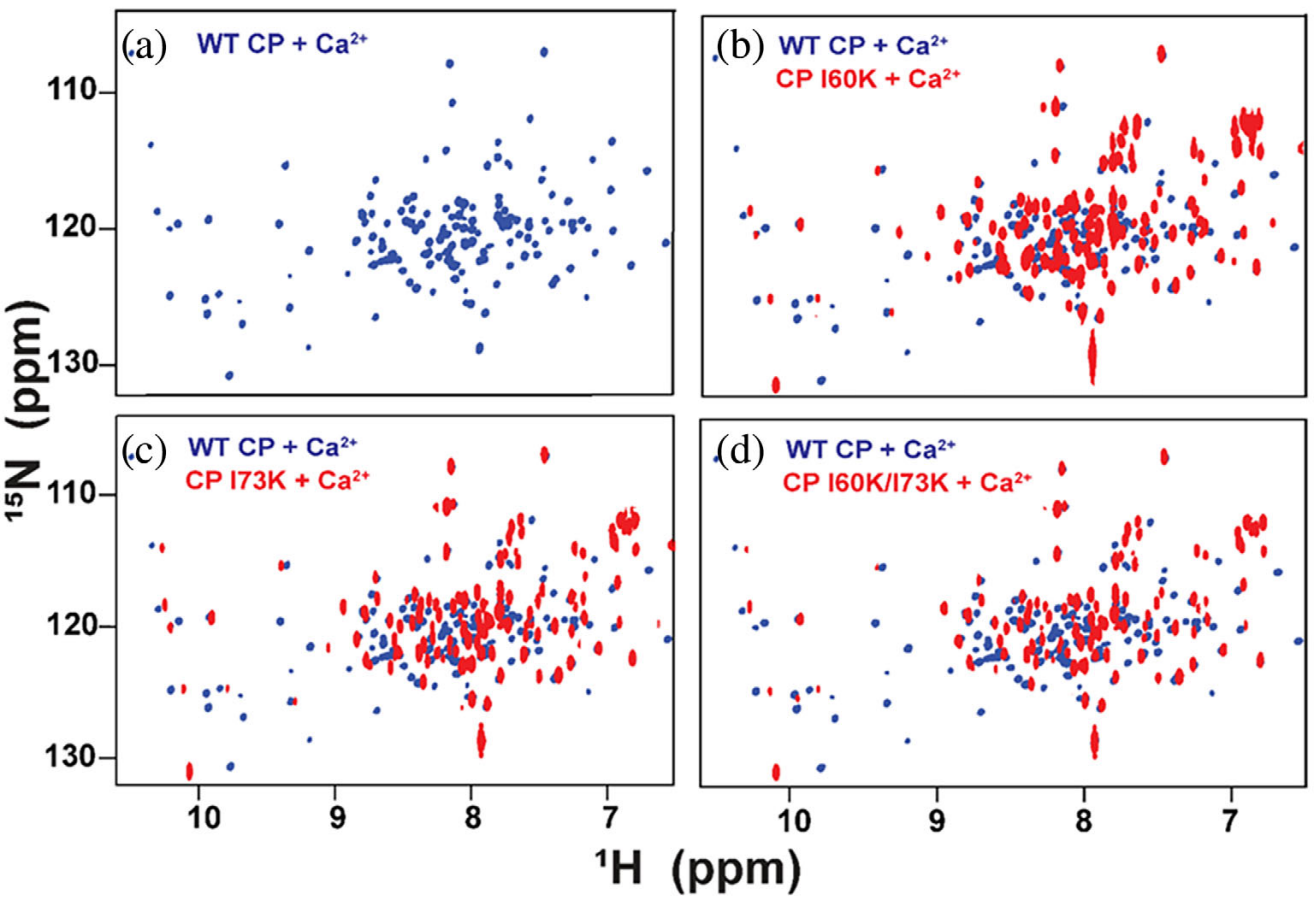
Calcium‐induced conformational changes of CP* and tetramerization‐deficient variants are similar. Two dimensional ^15^N‐^1^H NMR HSQC spectra of Ca^2+^‐loaded CP* and variants acquired at 900 MHz and 37°C. The spectra show well‐dispersed peaks with homogeneity in peak shape and intensity, as well as downfield signals characteristic of S100 proteins binding calcium. The spectrum for CP* was obtained from ^2^H,^15^N‐enriched protein and therefore has narrower linewidths than those of the variants.

As noted above, the addition of calcium to the WT protein resulted in substantial line broadening and corresponding loss of signal intensity in the spectrum as described above, consistent with the transition from the dimeric state in the absence of calcium to the tetrameric state upon binding of calcium (Figure 2). In contrast to CP*, the signals in the spectra of the three variants did not substantially broaden or lose intensity upon the addition of calcium. Moreover, the spectral changes were similar to those observed for other S100 proteins that have been characterized by NMR, including the characteristic appearance of peaks with ^1^H chemical shifts >9.5 ppm that arise from hydrogen bonds and salt bridges formed by residues in the calcium binding loops (Drohat et al., 1998; Harrison et al., 2023; Skelton et al., 1992). The NMR spectra obtained for the calcium‐loaded state of CP* and the three variants show that the calcium‐induced conformational changes in the tetramerization‐deficient variants mimic those observed for CP* (Figure 6). The similarity of the spectra obtained for I60K, I73K, and I60K/I73K shows that there are no significant structural differences between these three variants (Figure S2). Together, the chemical shift perturbations combined with the lack of significant broadening of the spectra suggest the CP* variants undergo the expected conformational changes induced by the binding of calcium while remaining in the dimeric state, that is, that the variant designs were successful.

To more directly characterize the oligomerization state by NMR, CPMG (Carr–Purcell–Meiboom–Gill) NMR experiments were performed, comparing CP* to the I60K/I73K variant. CPMG experiments provide a direct and simplified approach to obtaining information on protein dynamics using NMR relaxation parameters (Meiboom & Gill, 1958). The T_2_ parameter extracted from these experiments can be interpreted based on larger molecules (e.g., CP* tetramer) tumbling more slowly than smaller molecules (e.g., CP* dimer), which is reflected in more rapid loss of phase coherence and shorter T_2_ values. The significantly shorter T_2_ values measured for CP* (average of 0.03 s) versus I60K/I73K (average of 0.05 s) are consistent with the variant remaining dimeric in solution upon binding of calcium (Table S2).

### 2.5 The structure of calcium‐activated calprotectin is retained in CP* I73K

Multiple structures of calprotectin in various metal‐loaded states have been determined by x‐ray crystallography (Damo et al., 2013; Korndörfer et al., 2007; Nakashige et al., 2017). As part of our characterization of the CP* tetramer‐deficient variants, we pursued determination of their structures in the calcium‐loaded state in order to compare these to CP*. Interestingly, despite comprehensive screening efforts, high‐quality crystals of I73K were obtained using conditions that were similar to those used to crystallize CP*. The protein crystals diffracted to 1.7 Å and were crystallized in the P1,2_1_,1 space group with four heterotetramers in the asymmetric unit. The phasing of the data was determined by molecular replacement using the structure of calcium‐loaded CP* (PDB ID: 1XK4) as a search model. The final model was built and refined to an R_work_/R_free_ of 18%/21%. Overall, the tetramers were very similar to previously determined CP* structures. Moreover, the I73K heterodimer extracted from the tetramer is very similar to the heterodimers extracted from other CP* crystal structures. For example, the Cα RMSD from the Ca^2+^,Mn^2+^‐loaded CP* (PDB ID: 4GGF) is only 0.18 Å.

One noticeable feature of the structure was that although the crystallization condition did not contain any transition metals, even at the initial stage of refining the structure a metal ion was apparent in the His6 transition metal binding site. It turns out that the buffer contained MgCl_2_ and careful inspection of the reagent revealed it contained ~1% zinc (as well as lower concentrations of other transition metals). Given that CP* has a very high affinity for zinc, we assigned the metal ion as a zinc ion by iterative refinement, initially placing different transition metal ions in the His6 site and finding a zinc ion most agreed with the experimental data. We note that definitive assignment to zinc would require additional data collection at a higher wavelength than that of the Zn peak and comparison of the anomalous signals. The occupancy of the ion in the site was ~0.5 for all tetramers in the asymmetric unit. There was no zinc observed in the His3Asp site, which we attribute to the combination of only trace amounts of zinc being present and preferential crystallization with a Zn^2+^ ion in the His6 as opposed to the His3Asp site (Perera et al., 2025).

**FIGURE 7:**
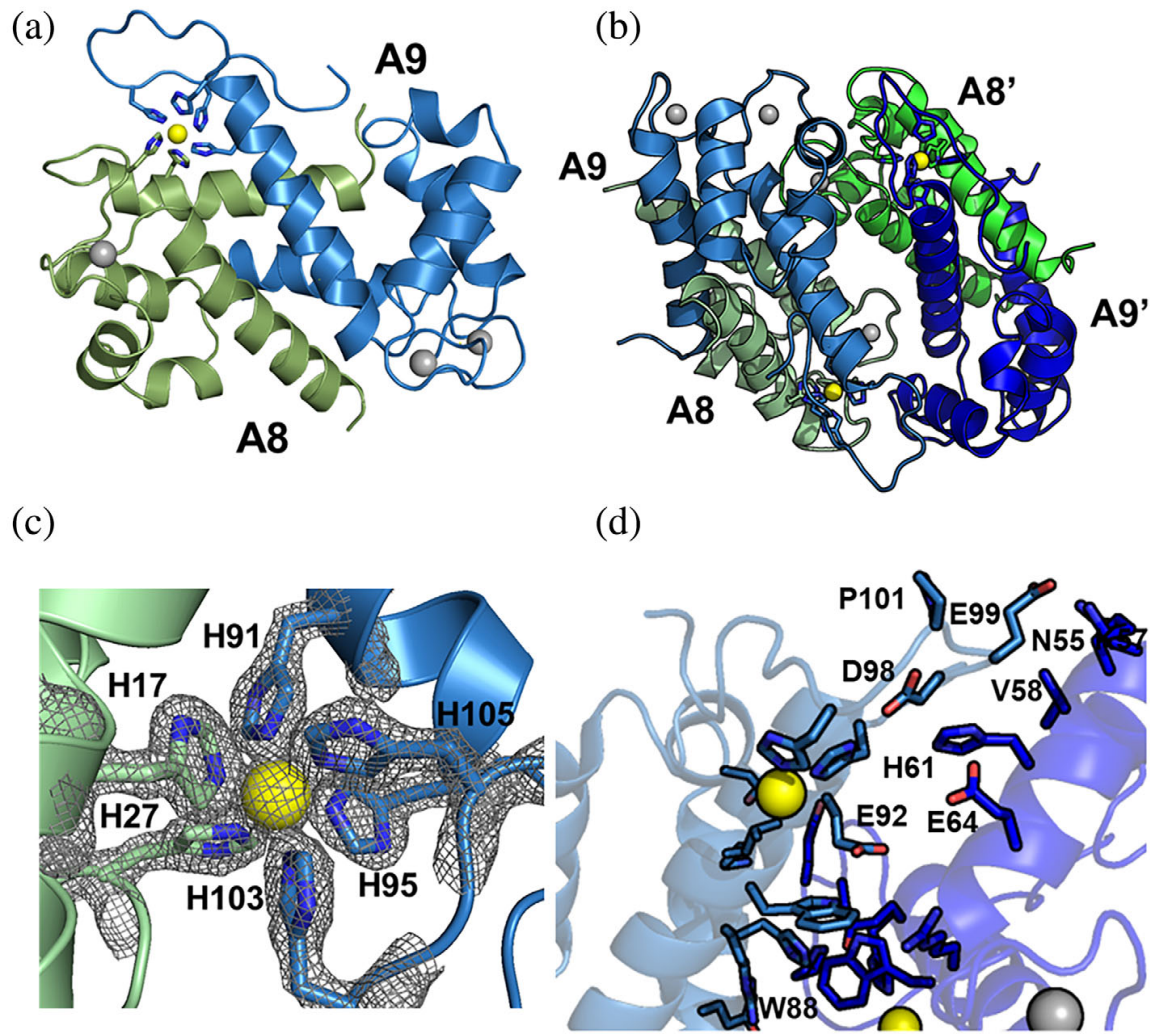
The I73K mutation does not alter the structure of CP*. (a) Ribbon diagram of I73K tetramer with S100A8 subunits colored green and S100A9 subunits blue. Calcium and zinc ions are depicted as gray and yellow spheres, respectively. (b) Zoomed in view of I73K His6 binding site with electron density (2F_O_‐F_C_ map contoured at 2σ) for the zinc ion and histidine side chains. (c) Overlay of I73K heterodimer extracted from the tetramer overlaid with the heterodimer extracted from the Mn^2+^‐loaded structure (PDB ID: 4GGF). Residues coordinating the zinc ion are shown as sticks. (d) I73K A9/A9′ interface with interfacing residue sidechains shown as sticks.

Whereas the overall secondary and tertiary structure of the I73K tetramer is very similar to the structure of Ca^2+^‐loaded CP*, the C‐terminal extension (tail) of S100A9 was not (Figure 7). Inspection of the Ca^2+^‐loaded CP* structure reveals weak to no density for the tail because there is no ion in the His6 site, suggesting that the tail is flexible. Density for the tail is observed when a metal ion is bound indicating the tail becomes more rigid, presumably because His103 and His105 from the tail coordinate the ion (Figure 7). In our structure of I73K, we found that the S100A9 tail forms crystal contacts not observed in the Ca^2+^‐loaded CP* structure and that the conformation of the tail is nearly identical to CP structures with an ion in the His6 site (e.g., the Cα RMSD from the Ca^2+^, Mn^2+^‐loaded CP* [PDB ID: 4GGF] is 0.095 Å). Analysis by the PBDePisa server reveals these contacts are primarily within the S100A9/S100A9′ interface with the tail interacting with Helix 3 of the opposite S100A9 subunit of the tetramer. This is reflected in an increase in buried surface area at the interface between S100A9 and S100A9′ from 84.6 to 471.5 Å^2^ when compared to the structure of calcium‐loaded CP*. This interface is stabilized by six hydrogen bonds and salt bridges. Even though the protein is clearly dimeric in solution, these contacts are presumably sufficient to stabilize I73K as a tetramer in the crystal. This implies that stabilization of the tetramer associated with these contacts is able to outweigh the destabilization of the tetramer arising from the mutation of the hydrophobic isoleucine side chain to the charged lysine side chain.

**FIGURE 8:**
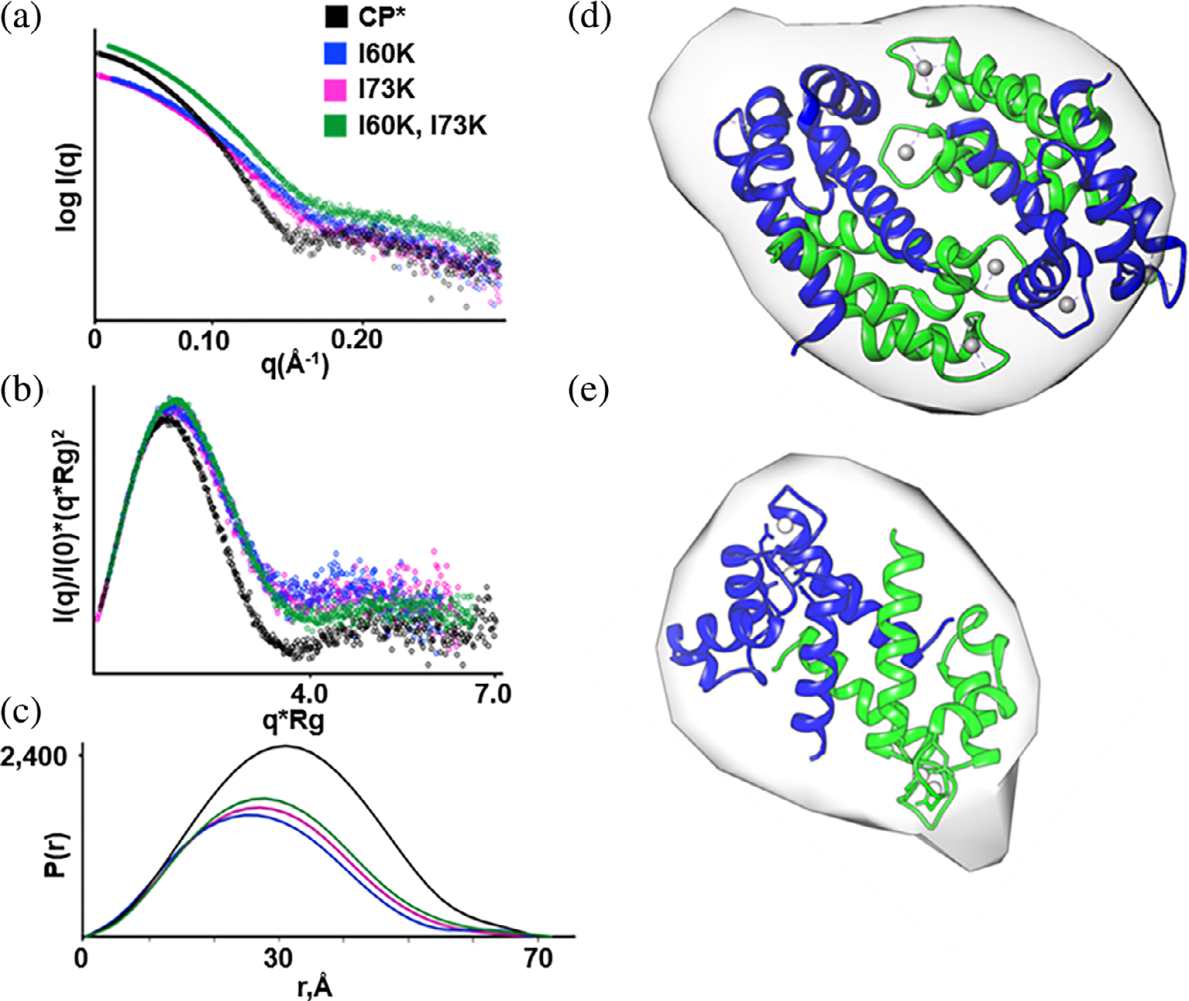
CP* forms a tetramer in solution but the tetramerization‐deficient variants do not. (a) Scattering profiles, (b) Kratky plots, and (c) P(r) functions for WT CP* and the three tetramer‐deficient variants. (d) Surface representation of the ab initio molecular envelope for WT CP* with the ribbon diagram of the calcium‐loaded CP* tetramer (PBD ID: 1XK4) fit into the envelope. (e) Surface representation of the ab initio molecular envelope for I73K with the ribbon diagram of a dimer extracted from the calcium‐loaded CP* tetramer crystal structure (PBD ID: 1XK4) fit into the envelope.

To test if the tetramerization of I73K induced by zinc binding was unique to the crystal structure and further assess the structures of the other CP* variants, we turned to small angle X‐ray scattering (SAXS). SAXS data were collected for CP* and the three variants under conditions of excess calcium (Figure 8). The data were of high quality overall. Analysis of the Guinier region of the log_10_ intensity plot indicates that all four samples were free of aggregation (Figure S3). Among the various parameters extracted from the SAXS data, the shape of the Kratky plots and high values of the Porod exponents (P_x_) for all four proteins reflect the well‐folded, globular nature of the protein. For CP*, the Kratky plot, R_g_, and D_max_ values from the P(r) function correlate well with Porod volumes, all consistent with a tetrameric state. In contrast, these parameters for the three variants indicate a globular dimeric state. Notably, the D_max_ values for all four proteins are very similar to the maximum interatomic distance corresponding to the length of one dimer, consistent with the near spherical shape of the tetramer (Table S3).

Porod exponents for all four proteins were close to four, which justifies direct analysis of the distance distribution function, P(r), to generate a molecular envelope. Ab initio shape calculations were performed using the density from solution scattering (DENSS) approach for all four proteins with the calcium‐loaded CP* structure (PDB ID: 1XK4) as the starting model. The average molecular envelope generated for CP* was fit well by tetramers extracted from our I73K crystal (Figure 8d). In contrast, the molecular envelopes generated for the three tetramer‐deficient mutants were substantially smaller than that of CP*, consistent with a dimeric state, and were well fit by heterodimers extracted from our I73K structure (Figure 8e). In summary, the SAXS data strongly support the hypotheses that the structure of calcium‐activated calprotectin is retained by the variants while remaining dimeric in solution even in the presence of excess calcium.

## 3 DISCUSSION

The work presented here builds on previously published studies of CP* I60K, which showed inhibition of tetramerization by disrupting hydrophobic contacts at the dimer‐dimer interface (Silvers et al., 2021; Stephan & Nolan, 2016). Our findings are consistent and extend the results reported by Silvers et al. Careful analysis of CP* crystal structures was used to design two additional tetramer‐deficient variants. A combination of DLS, NMR, and SAXS analysis of all three variants showed that the designs were stable and remained dimers in solution even under conditions of a 20‐fold excess of calcium. DLS revealed that the radius of CP* increases from just under 3 to ~3.4 nm as it binds calcium whereas the tetramerization‐deficient variants stay consistently under 3 nm, a radius that corresponds to a dimeric state. Basic ^15^N‐^1^H HSQC NMR experiments confirmed that the mutations did not significantly alter the structure of the protein and that all three variants undergo similar, significant conformational changes upon binding calcium. The I73K crystal structure showed that the overall secondary and tertiary structure is nearly identical to CP*. SAXS experiments confirmed that CP* is tetrameric in solution whereas the variants are dimeric. Overall, our results show that the design of tetramerization‐deficient variants was successful in that they disrupt calcium‐mediated tetramerization but no other physical properties of the protein. The formation of specific dimeric or tetrameric states at high levels of calcium appears to be a distinct characteristic of CP*, as other S100 proteins under conditions of excess calcium appear to transition into higher‐order oligomeric states (Moroz et al., 2009; Ostendorp et al., 2007).

The variants characterized in this work can be used as powerful reagents to test the biological relevance of calprotectin oligomerization. Although calprotectin mediates a number of intracellular functions, these variants will be most useful for studying the extracellular functions of calprotectin as secretion into the high calcium extracellular milieu triggers the switch from dimer to tetramer. Intracellular calcium levels are tightly regulated and maintained around 1 μM, insufficient to achieve the large excess of calcium we have shown to be required to drive tetramerization.

Our understanding of calprotectin oligomerization is further complicated by transition‐metal binding. The x‐ray crystal structure of CP* I73K shows this tetramerization‐deficient variant is susceptible to transition metal‐mediated oligomerization, as is CP* I60K, albeit at high concentrations of transition metal. It would be interesting to determine the minimum concentration required to induce CP oligomerization to help determine if the very low physiological concentrations of extracellular zinc are sufficient to induce oligomerization of tetramerization‐deficient variants. Regardless, the tetramerization‐deficient variants studied here provide useful tools for in vitro experiments, such as examining the interaction with receptors. Moreover, strategies to eradicate transition metal binding to calprotectin are well established (Damo et al., 2013), and the addition of such mutations could be used to generate variants that do not oligomerize in the presence of transition metals.

The molecular basis for calprotectin activation of its receptors remains largely unknown due to the absence of biophysical and structural information on interactions with any of its receptors. Biophysical characterization of calprotectin by NMR and protein crystallography is complicated by calcium‐mediated tetramerization. For example, NMR resonance assignments are available for more than 10 S100 proteins whereas there are none available for either subunit of calcium‐loaded calprotectin (Cristóvão et al., 2018; Penumutchu et al., 2013; Skelton et al., 1992). The additional challenges associated with the larger heterotetramer have led to functional analyses based on comparisons to S100A8 and S100A9 homodimers or variants that are unable to bind calcium and undergo the calcium‐induced conformational changes. While providing useful insights, the information obtained is fundamentally limited and has resulted in critical gaps in our knowledge of CP‐mediated signaling and pathology. Our tetramerization‐deficient variants provide the key reagents to directly address these gaps.

In summary, we have utilized an approach to comprehensively examine the oligomerization states of soluble proteins in solution. In addition, we have established separation of function variants that strictly modify tetramerization but no other physical properties of CP. These reagents can be used to better understand the physiological and pathological functions of calprotectin as well as facilitate future structural studies of CP‐receptor interactions, which will clarify mechanisms of CP‐mediated signaling and the broader implications of CP‐mediated inflammation.

## 4 MATERIALS AND METHODS

### 4.1 Expression plasmids

Site‐directed mutagenesis was used to generate the I60K and I73K expression plasmids. Expression vectors containing S100A8 C42S were mutated using a Q5 mutagenesis kit (New England Biolabs) with the primers listed below and annealing temperatures of 55°C–58°C (Hunter & Chazin, 1998). The pET1120 S100A8 I60K plasmid and primers designed for S100A8 I73K were used to make the corresponding double mutant. The DNA sequence of all plasmids was confirmed by Sanger sequencing.

**Table:** 

Primer	Sequence 5′ to 3′
I60K forward	CTGGTTCAAAGAGTTGGAT**AAA**AACACTGATGG
I60K reverse	ACGTCTGCACCCTTTTTCCTGATATACTG
I73K forward	CCAGGAGTTCCTC**AAA**CTGGTGATAAAGATG
I73K reverse	AAGTTAACTGCACCATCAGTGTTGATATCC

### 4.2 Protein expression and purification

The protocol for expression and purification of human calprotectin has been previously described (Hunter & Chazin, 1998). In order to avoid problems associated with the formation of non‐native disulfide bonds, we used the S100A8 (C42S) and S100A9 (C3S) plasmids to prepare CP* and the variants with mutations in the S100A8 (C42S) plasmid. CP* and CP* variants were prepared similarly (Hunter & Chazin, 1998; Kehl‐Fie et al., 2011). Chemically competent C41(DE3) cells were transformed with the relevant S100A8 and S100A9 plasmids. One liter cultures of Luria Broth (LB) were supplemented with 50 μg/mL ampicillin and grown at 37°C at 250 rpm until OD_600_ of approximately 0.8–1. Cultures were induced with 0.5 mM isopropyl β‐d‐1‐thiogalactopyranoside (IPTG) and the cells allowed to grow for 5 h at 37°C. Expression of ^2^H,^15^N‐enriched CP* was performed similarly, using M9 minimal medium in 99% D_2_O containing ^15^NH_4_Cl and ^2^H,^13^C‐glucose as the sole nitrogen and carbon sources. Starter cultures were adapted to 99% deuterated media by reaching an OD_600_ of 0.6–0.8 in 25%, 50%, and 75% deuterated media before 1 L cultures were inoculated. Cells were then harvested by centrifugation at 6000 rpm for 20 min and pellets were resuspended in buffer A (20 mM Tris–HCl pH 8.0, 100 mM NaCl, 1 mM EDTA) and combined. Cells were lysed by sonication and the cell debris removed by centrifugation at 12000 rpm for 20 min. The supernatant was discarded, and the pellet was resuspended in five times its weight with buffer A. Sonication and centrifugation steps were repeated on the inclusion bodies a total of two times prior to resuspension in buffer B (6 M Urea, 20 mM Tris–HCl pH 8.0, 100 mM NaCl, 1 mM EDTA). At this stage, the denatured S100A8 and S100A9 pellets were sonicated on ice and the solution clarified by centrifugation at 12000 rpm for 20 min. The supernatant was placed into dialysis tubing and refolded using three rounds of dialysis at 4°C in 20 mM Tris–HCl pH 8.0, 100 mM NaCl buffer. Precipitate was removed by centrifugation and filtration and loaded onto a HiTrap Q Sepharose FF anion exchange column. The protein was eluted using a NaCl gradient over 10 CV (0–300 mM). Fractions containing CP* were identified using SDS‐PAGE. The selected fractions were concentrated and loaded onto a HiPrep Sephacryl S‐200 HR size exclusion column in SEC Buffer (20 mM Tris–HCl pH 7.5, 100 mM NaCl). Fractions containing CP* were identified by SDS‐PAGE and stored at −80°C.

### 4.3 Isothermal titration calorimetry

Calprotectin binding affinity for RAGE VC1 was measured using a TA Instruments Affinity ITC. RAGE VC1 protein samples were used within 24 h of purification and samples of calprotectin were thawed from flash frozen aliquots. The calprotectin and RAGE VC1 samples were dialyzed into buffer containing 50 mM HEPES (pH 7.5), 150 mM NaCl, and 10 mM CaCl_2_ at room temperature for 2 h. All solutions were run through a 0.2 μm filter and extensively degassed before titration. Titrations were performed at 25°C with 125 rpm stirring and included an initial injection of 0.5 μL of 100 μM calprotectin into the sample cell containing 50 μM RAGE VC1, followed by an additional 23 injections of 2 μL each. Injections were spaced over 200–250 s intervals. Data were analyzed using TA Instruments NanoAnalyze and the thermodynamic parameters and binding affinities were calculated using the average of three titrations fit to an independent binding model.

### 4.4 Analysis of protein interfaces

The PDBePISA server was used to analyze the residues involved in stabilizing the CP* tetramer interface for calcium‐loaded CP* (PDB 1XK4) and the CP* I73K calcium and zinc‐loaded (PDB 9ON4) structures (Krissinel, 2010).

### 4.5 Dynamic light scattering

Prior to analysis aliquots stored at −80°C were thawed and diluted to 50 μM using buffer containing 20 mM Tris pH 7.5, 100 mM NaCl. Light scattering data were collected using a Wyatt DynaPro NanoStar instrument. Ten microliters of sample was loaded into Nanostar disposable microcuvettes of 1 cm pathlength. Protein was centrifuged at 12000 rpm at 4°C prior to measurement. All points are an average of 10 acquisitions and were measured three times.

### 4.6 Nuclear magnetic resonance

Samples were concentrated to 50–100 μM in a buffer containing 20 mM Tris, pH 7.5, 100 mM NaCl, and 5% ^2^H_2_O. Two identical solutions of protein were prepared for each sample, one with no calcium added and the other with 2 mM CaCl_2_. ^15^N‐^1^H HSQC spectra were recorded in 3‐mm tubes at 37°C using Bruker AVANCE 600 or 900 MHz spectrometers equipped with a TCI cryoprobe. ^15^N‐^1^H HSQC experiments were acquired with 64 scans, and 2048 points and 128 points in the direct and indirect dimensions, respectively. The data were initially processed using Topspin 3.6.4 (Bruker Biospin) then transferred to SPARKY for further processing and analysis (Lee et al., 2015). CPMG experiments to measure T_2_ were performed on a sample with a protein concentration of 100 μM with delay times of 0.017, 0.034, 0.051, 0.068, 0.102, 0.136, 0.170, and 0.204 s. These data were processed using Topspin Dynamics.

### 4.7 Small‐angle X‐ray scattering

The SAXS profiles for CP*, I60K, I73K and I60K/I73K were acquired on the SIBYLS beamline 12.3.1 at the Advanced Light Source. Data were collected in SEC‐SAXS mode, which uses additional inline instrumentation and detectors coupled to a size exclusion column. CP* concentration was adjusted to 2 mg/mL in 60 μL of SEC running buffer. The x‐ray wavelength was set to 1.127 Å and the sample‐to‐detector distance to 2100 mm. This combination gives scattering vectors (q) ranging from 0.01 to 0.4 Å^−1^. The scattering vector is q = 4πsinθ/λ, where 2θ is the scattering angle. The SAXS flow cell was coupled to an Agilent 1260 Infinity HPLC system using a Shodex KW‐802.5 SEC column equilibrated with SEC buffer at a flow rate of 0.65 mL/min. The eluent was subjected to the following: (1) ultra‐violet light (UV) at 280 nm, (2) multi‐angle light scattering (MALS), (3) quasi‐elastic light scattering (QELS), (4) SAXS, and (5) measurement of refraction. For SAXS measurements, 2 s x‐ray exposures were collected continuously during a 25 min elution. All frames for analyses had one SAXS frame corresponding to the running buffer before the detection of a peak subtracted from each.

The radius of gyration (Rg) was calculated for each of the subtracted frames using the Guinier approximation: I(q) = I(0) exp(−q^2^Rg^2^/3) with the limits qRg <1.3. The elution peak was compared to the integral of ratios to background and Rg relative to the recorded frame using the program RAW (Roh & Sohn, 2018). Uniform Rg values across an elution peak represent a homogeneous sample. Final merged SAXS profiles, derived by integrating multiple frames at the elution peak, were used for further analyses. We calculated the Guinier plot to provide information on the aggregation state, the volume of correlation (Vc) to estimate the molecular weight, and the pair distribution function [P(r)] to calculate the maximal inter‐particle dimension. Ab initio molecular envelopes were generated using DENSS (Grant, 2018). Visualization of the envelopes and superposition of models into them utilized the Chimera Fit to Map tool and DENSS align (Pettersen et al., 2004).

### 4.8 X‐ray crystallography

I73K was diluted to 1 mg/mL (47 μM) in a buffer containing 20 mM HEPES (pH 7.5), 100 mM NaCl, and 2 mM CaCl_2_, then concentrated to 10 mg/mL using centrifugation through a 10‐kDa membrane molecular weight cutoff. Crystals were obtained by the hanging drop vapor diffusion method using 0.1 M Bis‐Tris (pH 6.0), 0.2 M MgCl_2_, and 18% (mass/vol) PEG 3350. Crystals were vitrified in liquid nitrogen using the mother liquor supplemented with 25% (vol/vol) glycerol as a cryoprotectant. The crystals belonged to the primitive space group P 1 21 1, with unit cell dimensions of a = 106.74 Å, b = 83.78 Å and c = 109.91 Å. The final x‐ray diffraction data were collected at a wavelength of 0.978 Å (12,677 eV) on the LS‐CAT beamline 21‐ID‐G of the Advanced Photon Source at Argonne National Laboratory. Data were collected over 180° with a 0.2° oscillation per frame and processed with HKL2000 (Otwinowski & Minor, 1997). The data were phased by molecular replacement using the program Phaser with the coordinates for Ca^2+^, Mn^2+^‐bound CP* (PDB ID: 4GGF) as the search model (McCoy et al., 2007). Three tetramers, one dimer, and two subunits were found in the asymmetric unit. Several rounds of model building with Coot and refinement with Phenix were used to produce the final model (Emsley & Cowtan, 2004; Liebschner et al., 2019).

## AUTHOR CONTRIBUTIONS

**Velia Garcia:** Conceptualization; data curation; formal analysis; investigation; methodology; visualization; writing – original draft; writing – review and editing; validation. **Areetha D'Souza:** Data curation; formal analysis; investigation; validation; visualization; writing – review and editing. **Natalia Kozlyuk:** Data curation; formal analysis; investigation; validation; methodology; visualization. **Yasiru R. Perera:** Data curation; formal analysis; visualization; methodology. **Steven M. Damo:** Investigation; validation; formal analysis; methodology. **Walter J. Chazin:** Conceptualization; methodology; funding acquisition; formal analysis; data curation; investigation; project administration; resources; supervision; validation; visualization; writing – original draft; writing – review and editing.

## CONFLICT OF INTEREST STATEMENT

The authors declare no competing interests.

## Supporting information


**Figure S1.** CP* binds the VC1 domain of RAGE. Representative ITC titration and binding isotherm from integrated heat for the VC1 domain of RAGE with the addition of CP* in the presence of calcium. The mean and standard deviation K_d_ values were 377 ± 446 nM.
**Figure S2.** NMR HSQC overlay of CP* mutants. 900 MHz 2D ^15^N‐^1^H NMR HSQC overlay spectra of Ca^2+^‐loaded CP* variants. In order, I60K is in black, I73K is in red, and the I60K/73K in blue. The three variants are characteristic of calcium‐bound S100 proteins and overlay well with minimal perturbations across variants.
**Figure S3.** Guinier plots of the SAXS data acquired for CP* (black), I60K (blue), I73K (pink) and I60K/I73K (green). SAXS data was collected for CP* and mutants in the presence of 10‐fold excess calcium. The linearity of the Guinier plot shows that all four samples are free of aggregation in solution.
**Table S1.** PDBePisa analysis of CP* tetramer interfaces. Interacting residues are distinguished by different colors of the two chains. Residues that contribute to hydrogen bonds in the interface are underlined. Residues that contribute to salt bridges in the interface are marked with an asterisk.
**Table S2.** NMR spin–spin relaxation parameters of CP* and I60K/I73K extracted from CPMG experiments. ^15^N‐CP* and CP I60K/I73K were prepared as previously described. 100 uM of CP* and CP mutant was prepared in the presence of 10‐fold excess calcium. As assignments are not available for either protein peaks were selected by automatic peak picking using Topspin. 1/T_2_ values were determined for each peak visible in the 2D HSQC. Differences in number of visible peaks can be attributed to differences in concentration.
**Table S3.** Selected SAXS parameters for CP*, I60K, I73K, and I60K/I73K. High quality SAXS data were collected for CP* and CP mutants in the presence of 10‐fold excess calcium. SAXS data is consistent with a well‐ordered and globular structures as reflected in the Porod Exponent of nearly four for all proteins.
**Table S4.** Data collection and refinement statistics.

## Data Availability

The data that support the findings of this study are openly available in Protein Data Bank at https://www.rcsb.org/, reference number 9ON4.
