# Supplementary material for: Design and characterization of calprotectin tetramerization variants for probing the role of oligomerization in receptor activation
Source: Protein Sci. 2025 Dec 22;35(1):e70399. doi: 10.1002/pro.70399 (PMC12720418; doi:10.1002/pro.70399)
Supplement: Supplementary file 1 — Figure S1. CP* binds the VC1 domain of RAGE. Representative ITC titration and binding isotherm from integrated heat for the VC1 domain of RAGE with the addition of CP* in the presence of calcium. The mean and standard deviation Kd values were 377 ± 446 nM. Figure S2. NMR HSQC overlay of CP* mutants. 900 MHz 2D 15N‐1H NMR HSQC overlay spectra of Ca2+‐loaded CP* variants. In order, I60K is in black, I73K is in red, and the I60K/73K in blue. The three variants are characteristic of calcium‐bound S100 proteins and overlay well with minimal perturbations across variants. Figure S3. Guinier plots of the SAXS data acquired for CP* (black), I60K (blue), I73K (pink) and I60K/I73K (green). SAXS data was collected for CP* and mutants in the presence of 10‐fold excess calcium. The linearity of the Guinier plot shows that all four samples are free of aggregation in solution. Table S1. PDBePisa analysis of CP* tetramer interfaces. Interacting residues are distinguished by different colors of the two chains. Residues that contribute to hydrogen bonds in the interface are underlined. Residues that contribute to salt bridges in the interface are marked with an asterisk. Table S2. NMR spin–spin relaxation parameters of CP* and I60K/I73K extracted from CPMG experiments. 15N‐CP* and CP I60K/I73K were prepared as previously described. 100 uM of CP* and CP mutant was prepared in the presence of 10‐fold excess calcium. As assignments are not available for either protein peaks were selected by automatic peak picking using Topspin. 1/T2 values were determined for each peak visible in the 2D HSQC. Differences in number of visible peaks can be attributed to differences in concentration. Table S3. Selected SAXS parameters for CP*, I60K, I73K, and I60K/I73K. High quality SAXS data were collected for CP* and CP mutants in the presence of 10‐fold excess calcium. SAXS data is consistent with a well‐ordered and globular structures as reflected in the Porod Exponent of nearly four for all proteins. [file PRO-35-e70399-s001.docx]

**Supporting Information**

**
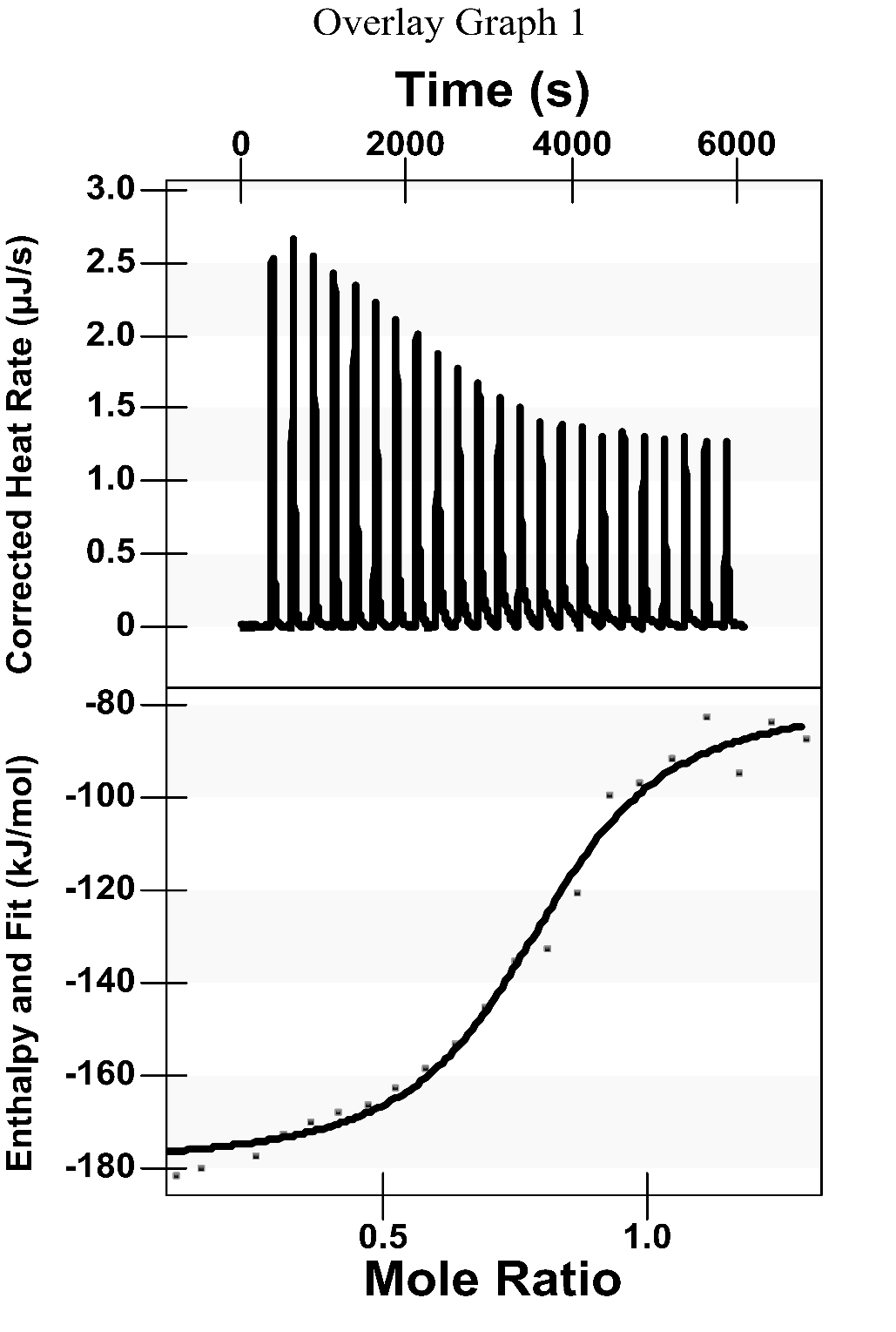
**

**Figure S1. CP* binds the VC1 domain of RAGE.** Representative ITC titration and binding isotherm from integrated heat for the VC1 domain of RAGE with the addition of CP* in the presence of calcium. The mean and standard deviation K_d_ values were 377 ± 446 nM.

**
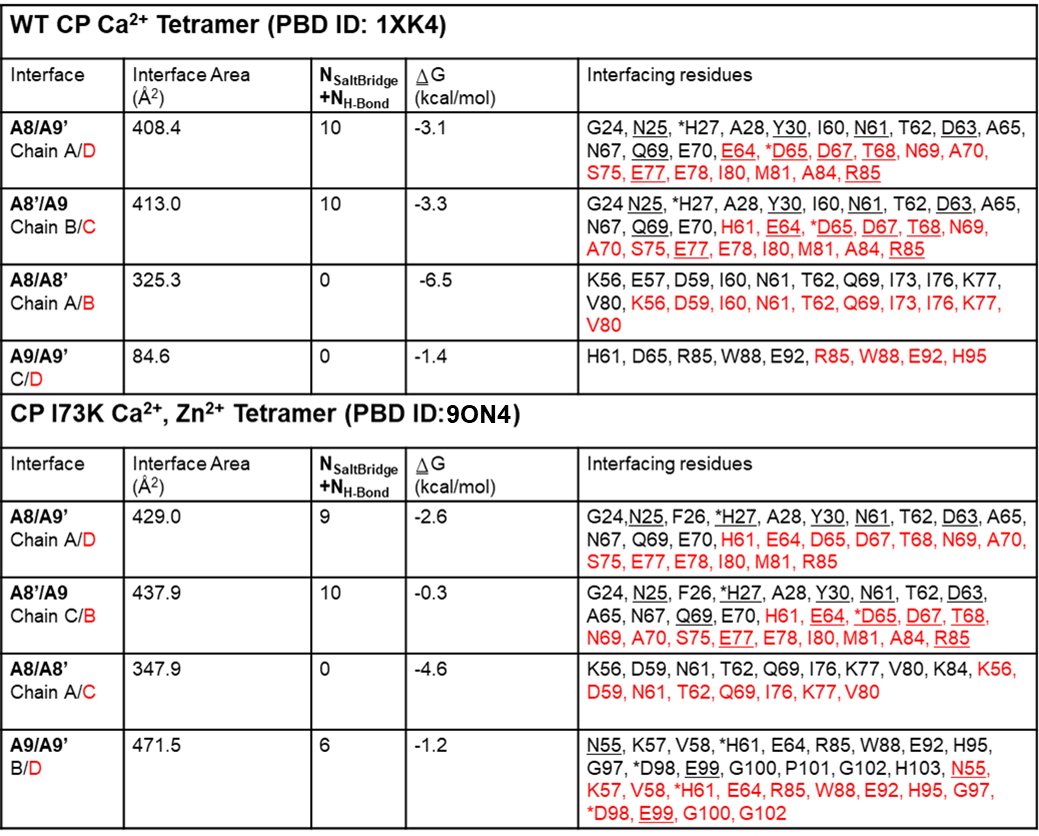
**

**Table S1. PDBePisa analysis of CP* tetramer interfaces.** Interacting residues are distinguished by different colors of the two chains. Residues that contribute to hydrogen bonds in the interface are underlined. Residues that contribute to salt bridges in the interface are marked with an asterisk.


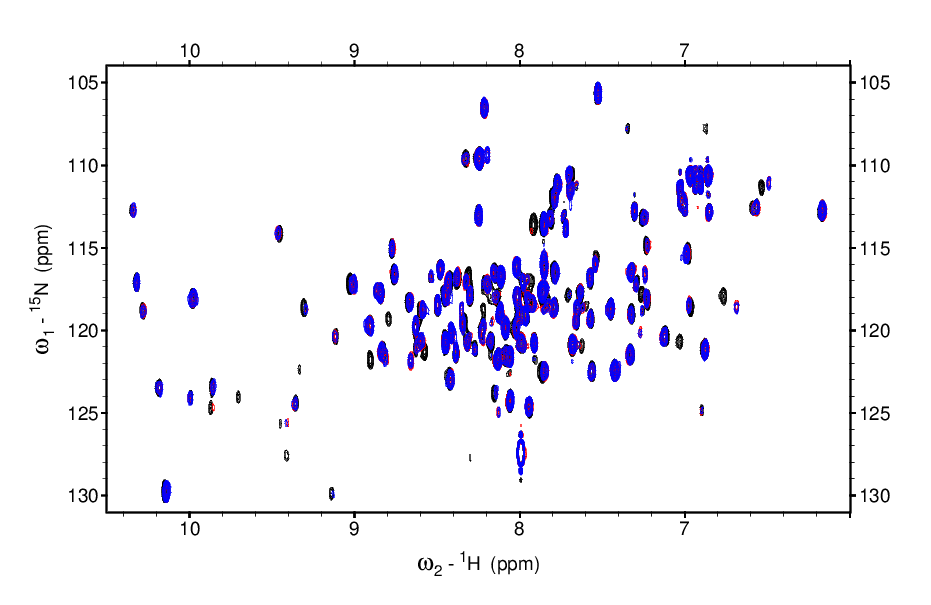


**Figure S2. NMR HSQC overlay of CP* mutants.** 900 MHz 2D ^15^N-^1^H NMR HSQC overlay spectra of Ca^2+^-loaded CP* variants. In order, I60K is in black, I73K is in red, and the I60K/73K in blue. The three variants are characteristic of calcium-bound S100 proteins and overlay well with minimal perturbations across variants.

**
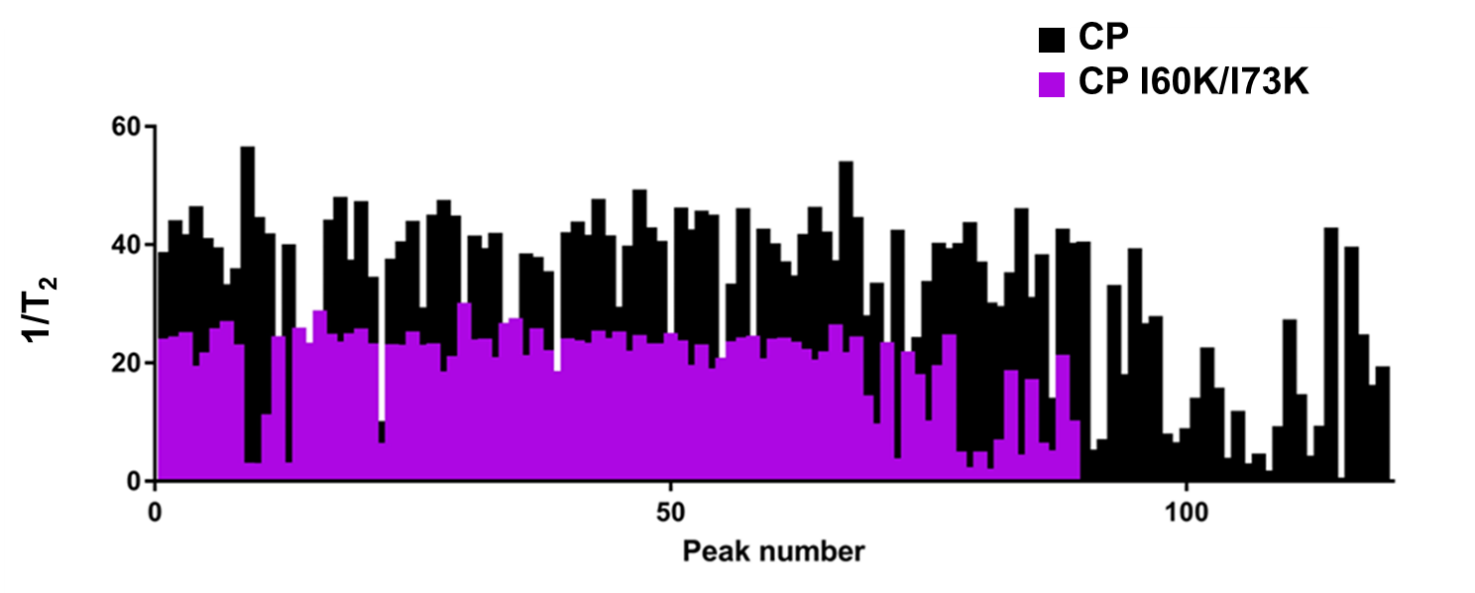
**

**Table S2. NMR spin-spin relaxation parameters of CP* and I60K,I73K extracted from CPMG experiments.** ^15^N-CP* and CP I60K/I73K were prepared as previously described. 100 uM of CP* and CP mutant was prepared in the presence of 10-fold excess calcium. As assignments are not available for either protein peaks were selected by automatic peak picking using Topspin. 1/T_2_ values were determined for each peak visible in the 2D HSQC. Differences in number of visible peaks can be attributed to differences in concentration.

**
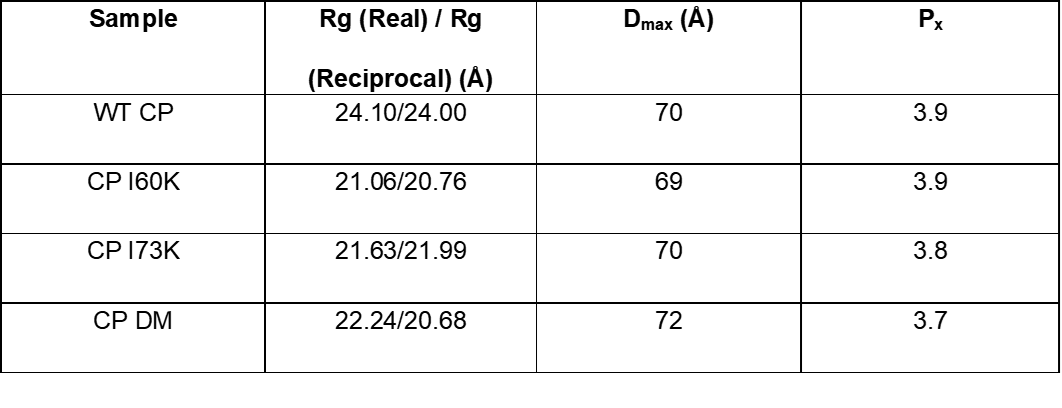
**

**Table S3. Selected SAXS parameters for CP*, I60K, I73K, and I60K/I73K.** High quality SAXS data were collected for CP* and CP mutants in the presence of 10-fold excess calcium. SAXS data is consistent with a well-ordered and globular structures as reflected in the Porod Exponent of nearly four for all proteins.

**Figure S3. Guinier plots of the SAXS data acquired for CP* (black), I60K (blue), I73K (pink) and I60K,I73K (green).** SAXS data was collected for CP* and mutants in the presence of 10-fold excess calcium. The linearity of the Guinier plot shows that all four samples are free of aggregation in solution.

**Table 4. Data collection and refinement statistics.**

|  | **CP I73K + Ca^2+^, Zn^2+^** |
| --- | --- |
| **Data Collection** |  |
| Wavelength | 0.97857 Å |
| Resolution, Å | 36.23 - 1.664 (1.71 - 1.66) |
| Space group | P1,21,1 |
| Total reflections | 858058 (66111) |
| Cell dimensions  *a, b, c,* Å  α, β, γ, ° | 106.48, 83.585, 109.685  90, 97.74, 90 |
| Unique reflections | 217299 (16427) |
| Multiplicity | 3.9 (4.0) |
| Completeness (%) | 93.47 (79.41) |
| Mean I/sigma(I) | 10.00 (0.96) |
| Wilson B-factor | 25.04 |
| R-merge | 0.0822 (1.087) |
| R-meas | 0.09498 (1.255) |
| R-pim | 0.04678 (0.6167) |
| CC1/2 | 0.995 (0.336) |
| CC* | 0.999 (0.709) |
| **Reflections used in refinement** | 208313 (13528) |
| Reflections used for R-free | 1831 (135) |
| R_work_ | 0.1838 (0.2437) |
| R_free_ | 0.2189 (0.2737) |
| Number of non-hydrogen atoms | 13756 |
| Protein | 12578 |
| Ligand/ion | 36 |
| Water | 1142 |
| Protein residues | 1569 |
| RMS(bonds) | 0.005 |
| RMS(angles) | 0.74 |
| **Ramachandran favored (%)** | 99.48 |
| Ramachandran allowed (%) | 0.52 |
| Ramachandran outliers (%) | 0.00 |
| Rotamer outliers (%) | 0.15 |
| Clashscore | 0.97 |
| **Average B-factor** | 29.15 |
| Protein | 28.59 |
| Ligand/ion | 24.77 |
| Water | 35.48 |

***Statistics for the highest-resolution shell are shown in parentheses.**
